# Oxygenation/non-invasive ventilation strategy and risk for intubation in immunocompromised patients with hypoxemic acute respiratory failure

**DOI:** 10.18632/oncotarget.26069

**Published:** 2018-09-14

**Authors:** Guillaume Dumas, Sylvie Chevret, Virginie Lemiale, Frédéric Pène, Alexandre Demoule, Julien Mayaux, Achille Kouatchet, Martine Nyunga, Pierre Perez, Laurent Argaud, François Barbier, François Vincent, Fabrice Bruneel, Kada Klouche, Loay Kontar, Anne-Sophie Moreau, Jean Reignier, Laurent Papazian, Yves Cohen, Djamel Mokart, Elie Azoulay

**Affiliations:** ^1^ Medical Intensive Care Unit, Saint-Louis Teaching Hospital, Paris, France; ^2^ ECSTRA Team, Biostatistics and Clinical Epidemiology, UMR 1153 (Center of Epidemiology and Biostatistic Sorbonne Paris Cité, CRESS), INSERM, Paris Diderot University, Paris, France; ^3^ Medical Intensive Care Unit, Cochin Teaching Hospital, Paris, France; ^4^ Medical Intensive Care Unit, Pitié-Salpêtrière Teaching Hospital, Paris, France; ^5^ Medical Intensive Care Unit, Angers Teaching Hospital, Angers, France; ^6^ Medical Intensive Care Unit, Victor Provo Hospital, Roubaix, France; ^7^ Medical Intensive Care Unit, Brabois University Hospital, Nancy, France; ^8^ Medical Intensive Care Unitt, Edouard Herriot Teaching Hospital, Lyon, France; ^9^ Medical Intensive Care Unit, La Source Hospital-CHR Orleans, Orléans, France; ^10^ Intensive Care Unit, CHI Le Raincy-Montfermeil, Le Raincy-Montfermeil, France; ^11^ Intensive Care Unit, Hopital Andre Mignot-Le Chesnay, Paris, France; ^12^ Intensive Care Unit, Lapeyronie University Hospital, Montpellier, France; ^13^ Centre Hospitalier Universitaire-Amiens, Amiens, France; ^14^ Centre de Réanimation, CHRU Lille, Lille, France; ^15^ Réanimation Médicale, Centre Hospitalier Universitaire-Nantes, Nantes, France; ^16^ Réanimation DRIS, Hôpital Nord, Marseille, France; ^17^ Intensive Care Unit, Hôpital d'Avicenne, APHP, Bobigny, France; ^18^ Intensive Care Unit, IPC, Marseille, France

**Keywords:** acute respiratory failure, immunocompromised, oxygenation, noninvasive ventilation, high flow nasal cannula

## Abstract

We investigated how the initial ventilation/oxygenation management may influence the need for intubation on the coming day in a cohort of immunocompromised patients with acute hypoxemic respiratory failure (ARF).

Data from 847 immunocompromised patients with ARF were used to estimate the probability of intubation at day+1 within the first 3 days of ICU admission, according to oxygenation management. First, noninvasive ventilation (NIV) was compared to oxygen therapy whatever the administration device; then standard oxygen was compared to High Flow Nasal Cannula therapy alone (HFNC), NIV alone or NIV+HFNC. To take into account the oxygenation regimens over time and to handle confounders, propensity score weighting models were used.

In the original sample, the probability of intubation at day+1 was higher in the NIV group vs oxygenation therapy (OR = 1.64, 95CI, 1.09–2.48) or vs the standard oxygen group (OR = 2.05, 95CI: 1.29–3.29); it was also increased in the HFNC group compared to standard oxygen (OR = 2.85, 95CI: 1.37–5.67). However, all these differences disappeared by handling confounding-by-indication in the weighted samples, as well as in the pooled model. Note that adjusted OR for day-28 mortality increased with the day of intubation.

In this large cohort of immunocompromised patients, ventilation/oxygenation management had no impact on the probability of intubation on the coming day.

## INTRODUCTION

The number of patients living with immune deficiency is increasing steadily [1, 2]. Despite major therapeutic advances [3, 4], these patients may experience several life-threatening complications, mostly acute respiratory failure (ARF), that require admission to the intensive care unit (ICU) [5].

ARF is associated with high mortality rates [6, 7]. When intubation is needed, case fatality is even significantly higher [5, 8, 9]. Hence, avoiding intubation and conventional mechanical ventilation (MV) has become a priority [10]. In that setting, use of noninvasive ventilation (NIV) or High Flow Nasal cannula therapy (HFNC) has been reported to be associated with decreased mortality [11, 12]. However, conflicting data have been reported [13, 14]. Noteworthy, the increasing popularity of these techniques has to be balanced with the risk of delayed intubation, which has been associated with increased mortality [15, 16].

As the literature remains not conclusive, guiding initial oxygenation and ventilation management in immunocompromised patients with ARF requires additional data. To address this clinical question and assist decision-making, we applied a propensity score based approach that allows handling potential time-dependent confounders, on a large cohort of immunocompromised patients with ARF. This study seeks to guide daily clinical decisions about oxygenation and ventilation management in patients who are not intubated on the first ICU day. This applies to up to 80% of immunocompromised patients with hypoxemic ARF. In other words, we sought to address the clinical question of whether prolonging NIV or high-flow oxygen beyond day-1 can actually decrease the proportion of patients further intubated [13, 19].

## RESULTS

### Patients

As shown in Supplementary Figure 1, a total of 1,121 immunocompromised patients were admitted in the participating ICUs for ARF. Among them, 847 patients who met the inclusion criteria, were analyzed in the present study. Table 1 reports their main characteristics at ICU admission. The main cause of immunosuppression was hematologic malignancy (*n* = 700, 83%) followed by solid cancer (*n* = 97, 11%), immunosuppressive drugs (*n* = 50, 6%), and solid organ transplant in 18 cases (2%). One hundred and thirty-seven patients (16%) underwent allogeneic stem cell transplantation. Acute leukemia (*n* = 262, 37.4%) and lymphoma (*n* = 211, 30.1%) were the most frequent underlying diseases. Malignancy was active (recent chemotherapy or ongoing) in 566 patients (71%). One hundred and seventeen patients (14%) had a poor Performance Status in the month before ICU admission. One third of patients had neutropenia. Median time from hospital admission to ICU requirement was 2 [IQR, 0–12] days and 324 (38.3%) were admitted directly from emergency department. At ICU admission, the respiratory symptoms evolved since 1 day [0–3]. The most common ARF etiology was infection (426 patients, 50%).

**Table 1:** Patients characteristics admitted with acute respiratory failure in ICU

Variables	Patients *N* = 847 (%)	No. Missing
Age, median (IQR), years	62 [52–70.5]	
Female sex	385 (45)	
Underlying disease		
Hematologic malignancies	700 (83)	
Solid tumors	97 (11)	
Immunosuppressive drugs	50 (6)	
Allogeneic stem cell transplantation	137 (16)	
Chronic hematologic malignancy	307 (36)	
Remission of the malignancy	231 (27)	
Major comorbidities		
Respiratory comorbidity^a^	246 (29)	
Chronic kidney insufficiency	89 (11)	
Cardiovascular diseases	351(41)	
Diabetes	117 (14)	89
HIV infection (with another immunosuppressive condition)	10 (1.2)	
Performans status >2	117 (14)	30
Body mass index at admission, median (IQR), Kg/m^2^	24.4 [21.8–27.3]	85
Neutropenia at ICU admission	230 (27)	
Direct ICU admission	324 (38.3)	
Time from hospital admission to ICU, median (IQR), days	2 [0–12]	
Time from first respiratory symptoms to ICU, median (IQR), days	1 [0–3]	
ARF etiology		
Infection	426 (50)	
Bacterial or viral pneumonia	276 (33)	
Opportunistic germs infection^b^	120 (14)	
Neutropenia recovery	83 (10)	
Malignant infiltration	84 (10)	
Others^c^	155 (18)	
No identified etiology	147 (17)	
SOFA score at admission, median (IQR)	5 [3–7]	60
Shock at admission	113 (13)	
Acute kidney injury at admission	328 (39)	7
Oxygen flow at ICU admission, median (IQR), L/min	8 [4–15]	
Maximum respiratory rate on Day 0, median (IQR)	32 [27–38]	48
Arterial blood gas at admission		
pH	7.44 [7.4–7.48]	
PaCO2, median (IQR), mmHg	34 [30–40]	
PaO2 median (IQR), mmHg	78 [64–100]	
PaO2: FiO2 ratio	127.8 [78.89–205]	
No. of lung quadrants with radiographic infiltrates	2 [1–4]	
ICU length of stay, median (IQR), days	5 [3–11]	
Invasive mechanical ventilation	268 (32)	
Time from admission to intubation, median (IQR), days	2 [1–4]	
Length of mechanical ventilation, median (IQR), days	7 [3–15]	
28-day mortality	181 (21)	
90-day mortality	295 (35)	

IQR: interquartile range, ARF: acute respiratory failure, SOFA: Sequential Organ Failure Assessment, ICU: intensive care unit, PaO2: partial pressure of arterial oxygen, PaCO2: partial pressure of arterial carbon dioxide, FiO2: fraction of inspired oxygen (measured or estimated in patients with standard oxygen therapy: oxygen flow (L/min) ^*^0.03 + 0.21.

^a^Including COPD in 52 (6%) patients.

^b^including: pneumocystis jirovecii pneumonia (*n* = 68), fungi (*n* = 51).

^c^including: extra pulmonary ARDS (*n* = 58), toxic pneumonia (*n* = 20), intra–alveolar hemorrhage (*n* = 7).

Overall, MV rate was 32% (*n* = 268 patients) with a median time between admissions to mechanical ventilation of 2 [1–4] days. Supplementary Figure 2 provides the cumulative incidence of mechanical ventilation requirement until day-14 according to each study. As shown, 78.6% (*n* = 187) of MV occurred during the first three ICU days. As reported in Table 1, the median ICU length of stay was 5 [3–11] days. The crude Day-28 mortality rate was 21.0% (181 deaths). In patients undergoing MV, it reached 58.5% (151 deaths). No significant difference in the cumulative incidence of MV between the different studies was observed (*p* = 0.44, see Supplementary Figure 2). Similarly, there was no evidence across the three studies of any difference in mortality rate in mechanically ventilated patients (*p* = 0.14).

### Oxygenation strategy and outcome

#### Model 1: Noninvasive ventilation vs. oxygen

#### Oxygenation strategy

Over the first three ICU days, 429 patients in the combined cohort, were treated by at least one NIV trial and 543 received at least one-day of oxygen. The marginal distribution of oxygenation strategies and the different sequences of oxygenation strategies that applied to patients consecutively during the first week of ICU, illustrated that patients may undergo different strategies through the ICU course (see Supplementary Figure 3A and 3B). The main parameters of oxygenation devices during the three first ICU days, are summarized in Table 2.

**Table 2:** Main characteristics of oxygenation support during the first three days of ICU (Model 1 and 2)

Variables	Day 0	Day 1	Day 2
Model 1			
**Noninvasive ventilation group**			
Number	381	284	223
Number of trial/day, median (IQR)	6 [3–6]	5 [3–6]	4 [2–6]
Length of NIV (h), median (IQR)	4.5 [2–7]	5 [3–8]	4 [3–7.5]
PEEP (cm H2O), median (IQR)	5 [5–6]	5 [5–6]	5 [4–6]
Pressure support (cm H2O), median (IQR)	8 [7–10]	8 [7–12]	8 [7–10]
FiO2 (%), median (IQR)	80 [50–95]	50 [32–90]	60 [36–95]
Gas flow between NIV sessions (L/min), median (IQR)	9.5 [4.8–15]	8 [5–15]	9 [4–15]
**Oxygen group**			
Number	466	379	279
Gas flow (L/min), median (IQR)	6 [3–12]	5 [2–9]	4 [2–8]
**Model 2**			
**Noninvasive ventilation group**			
Number	**329**	**242**	**191**
Number of trial/day, median (IQR)	6 [2–6]	5 [3–6]	4 [2–6]
Length of NIV (h), median (IQR)	5 [2–7]	5.5 [3–8]	4.25 [3–8]
PEEP (cm H2O), median (IQR)	5 [5–6]	5 [5–6]	5 [4–6]
Pressure support (cm H2O), median (IQR)	8 [6–10]	9 [7–12]	8 [7–10.5]
FiO2 (%), median (IQR)	80 [40–95]	80 [40–95]	55 [36–95]
Gas flow (L/min), median (IQR)	9 [4–15]	7 [4–11.5]	6 [4–12]
**High flow nasal cannula group**			
Number	**64**	**58**	**39**
FiO2 (%), median (IQR)	80[52.5–95]	70 [50–95]	67.5 [50–95]
Gas flow (L/min), median (IQR)	40 [27.5–50]	40 [30–50]	75 [50–95]
**Noninvasive ventilation with high flow nasal cannula group**			
Number	**52**	**42**	**32**
Number of trial/day, median (IQR)	6 [6–6]	6 [4–6]	6 [5–6]
Length of NIV (h), median (IQR)	4 [1.25–6]	4 [3–6]	4 [3–6]
Pressure support (cm H2O), median (IQR)	8 [7–10]	9 [8–12]	9 [8–11]
PEEP (cm H2O), median (IQR)	5 [4–6]	5 [4–6]	5 [4–6]
Gas flow (L/min), median (IQR)	40 [30–50]	40 [30–50]	40 [30–50]
FiO2 (%), median (IQR)	70 [50–95]	60 [45–90]	40 [30–50]
**Standard oxygen group**			
Number	**402**	**321**	**240**
Gas flow (L/min), median (IQR)	6 [4–15]	5 [4–11]	6 [3–9]

IQR: interquartile range, NIV: Noninvasive ventilation, HFNC: high flow nasal cannula, FiO2: fraction of inspired oxygen.

### Probabilities of mechanical ventilation requirement

Covariate balances before and after weighting, are reported in the Supplementary Materials. As shown in Table 3, using standard logistic regression on the original sample, the NIV use at day 0 was significantly associated with mechanical ventilation (crude OR 1.64, 95CI: 1.09–2.48; *p* = 0.02). On the weighted samples, no significant difference between NIV and oxygen group on the occurrence of mechanical ventilation on the coming day was observed, whatever the day of exposure. Based on the pooled model, the overall OR for mechanical ventilation requirement did not differ significantly between the two groups (*p* = 0.56, Table 3). Note that prevalence of mechanical ventilation differed over time.

**Table 3:** Estimated effect of ventilation/oxygenation strategy on mechanical ventilation requirement in the coming day according to stratified approach on landmark time and Structural Marginal Model (Model 1 and 2)

	Original sample		Weighted sample	
	Odds ratio (95% CI)	*P value*	Odds ratio (95% CI)	*P value*
**Landmark time-estimated effect**				
**Model 1**				
Day 1^*^				
Noninvasive ventilation at day 0	1.64 (1.09–2.48)	0.019	1.01 (0.96–1.07)	0.657
**Day 2**				
Noninvasive ventilation at day 1	1.31 (0.75–2.31)	0.341	1.01 (0.96–1.06)	0.827
**Day 3**				
Noninvasive ventilation at day 2	1.46 (0.70–3.10)	0.125	1.01 (0.95–1.06)	0.812
**Model 2**				
Day 1^*^				
Noninvasive ventilation at day 0	2.05 (1.29–3.29)	0.002	1.02 (0.98–1.07)	0.307
High Flow-nasal cannula at day 0	2.85 (1.37–5.67)	0.004	1.10 (0.95–1.28)	0.211
NIV with HFNC at day 0	1.74 (0.67–3.96)	0.214	1.00 (0.92–1.10)	0.933
**Day 2**				
Noninvasive ventilation at day 1	1.50 (0.82–2.75)	0.191	0.99 (0.96–1.03)	0.734
High Flow-nasal cannula at day 1	1.28 (0.42–3.29)	0.631	0.98 (0.91– 1.06)	0.670
NIV with HFNC at day 1	0.68 (0.11–2.43)	0.610	0.96 (0.92– 1.01)	0.127
**Day 3**				
Noninvasive ventilation at day 2	1.65 (0.73–3.80)	0.230	1.00 (0.96–1.05)	0.822
High Flow-nasal cannula at day 2	1.73 (0.38–5.88)	0.415	0.97 (0.94–0.99)	0.008
NIV with HFNC at day 2	1.39 (0.21–5.50)	0.679	1.04 (0.90–1.20)	0.594
**Marginal structural model–estimated effect**				
**Model 1**				
Noninvasive ventilation	_		1.10 (0.78–1.54)	0.562
Onset of oxygenation strategy at day 0	_		0.57 (0.39–0.84)	0.004
Onset of oxygenation strategy at day 1	_		0.43 (0.27–0.69)	0.004
**Model 2**				
Noninvasive ventilation	_		1.07 (0.81–1.62)	0.455
High flow nasal cannula	_		1.14 (0.65–3.41)	0.345
NIV with HFNC	–		1.00 (0.41–2.44)	0.992
Onset of oxygenation strategy at day 0	_		0.58 (0.58–1.90)	0.011
Onset of oxygenation strategy at day 1	_		0.51 (0.50–0.82)	0.049

NIV: Noninvasive ventilation, HFNC: high flow nasal cannula.

^*^Day since admission (Day 0).

**Figure 1:**
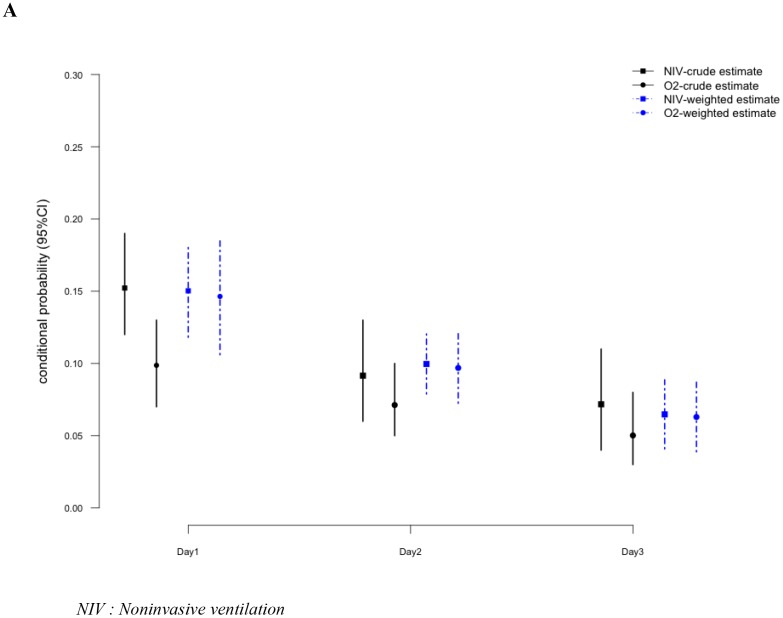

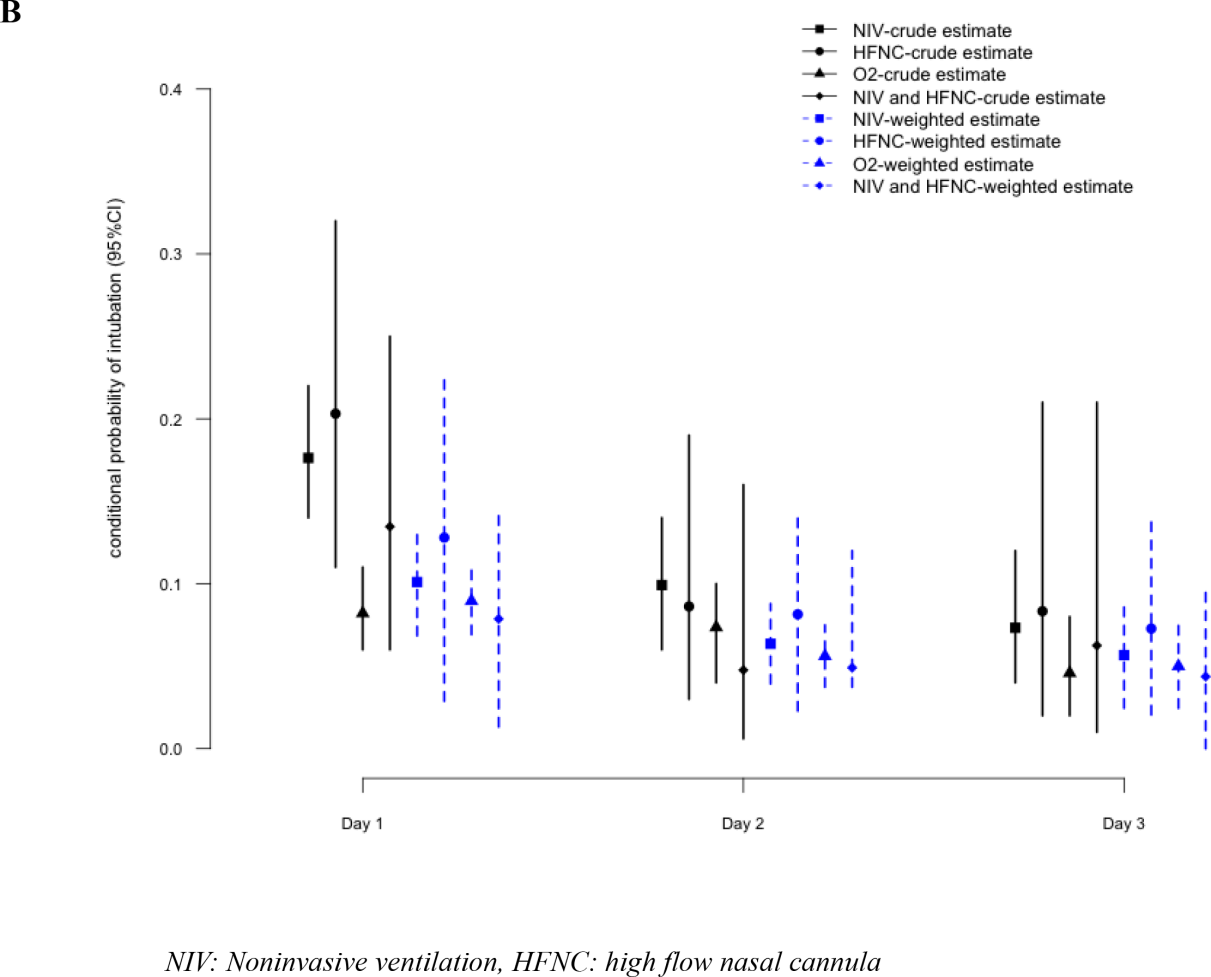
Estimated probability of Mechanical ventilation on the coming day according to the current oxygenation strategy - Model 1 (**A**), Model 2 (**B**).

We further showed no evidence of any interaction between day of exposure and NIV effect (*p* = 0.97). These results are illustrated in the Figure 1A that provides the estimated conditional probability of intubation on the coming day according to the oxygenation strategy choice, in the original sample and in those obtained with the pooled model. The crude estimated probability of MV at day +1 was 15.2% (95 CI: 11.7–19.2) in the NIV group, and 9.8% (95 CI: 0.07–12.9) in the oxygen group. After weighting, these probabilities were close, with a MV probability estimate of 14% (95 CI: 18–10) in the oxygen group and 15% (95 CI: 12–18) in the NIV group.

### Model 2: Standard oxygen alone vs. NIV alone, NIV+HFNC or HFNC alone

#### Oxygenation strategy

During the first three days of ICU, 488 patients received standard oxygen therapy, 378 patients received NIV alone, 60 patients both NIV and HFNC, and 89 patients received HFNC alone. Supplementary Figure 4A and 4B display the different distributions of oxygenation states and trajectories during the first six days in ICU, respectively. Table 2 provides the main features of the three different oxygenation strategy groups during the first three days in ICU. At admission, patients treated with standard oxygen alone, received oxygen at a median flow of 6 [4–15] L/min. In the NIV group, median NIV duration was 4.5 [2–7] hours within the first 24 hours, 5 [3–8] hours on day 2, and 4 [3–7.5] hours on day 3. In HFNC group, the gas flow was 40 [27.5–50] L/min the day of admission with a fraction of inspired oxygen (FiO2) at 80% [52.5–95].

### Probabilities of mechanical ventilation requirement

Covariates balances before and after weighting are reported in the Supplementary Data. Figure 1B displays the prevalence of intubation on the coming day from admission to day 2, in the original and weighted samples, and Table 3 reports the estimated effect of oxygenation strategy at each of these days in both datasets. Using standard logistic estimation on the original sample, OR for intubation at day 1 was significantly higher in the NIV group (OR: 2.05, 95 CI: 1.29–3.29) and HFNC group (OR: 2.85, 95 CI: 1.37–5.67) than those in the standard oxygen group. These differences were no longer significant in the weighted samples. Using pooled model, we observed no effect of the oxygenation strategy on intubation, regardless of the oxygenation devices (Table 3). As above, time was associated with a reduced occurrence of mechanical ventilation (day 1: OR: 0.58, 95 CI 0.58–1.9; day 2: OR: 0.51, 95 CI: 0.50–0.82), as compared to day 0.

### Sensitivity analyses

We did not find any heterogeneity in the ventilation effect according to oxygenation strategy, between patients with and without determined ARF etiology (model 1: *p* = 0.86, model 2: *p* = 0.66).

### Mortality within 28 days

**Figure 2:**
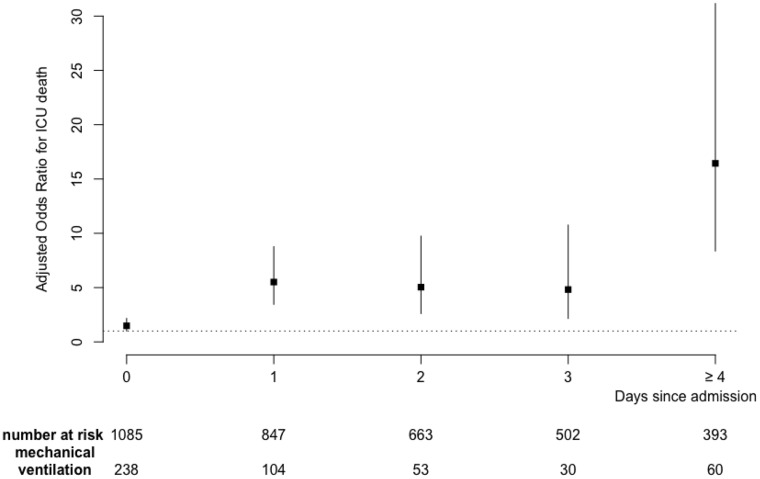
Adjusted odds ratio for relationship between the day of invasive mechanical ventilation initiation and Day-28 mortality Results were adjusted on SOFA, Age and Performans status at admission.

Figure 2 illustrates the relationship between the day of invasive mechanical ventilation initiation and day-28 mortality regardless the oxygenation/ventilation strategy. After adjustment on SOFA at admission, Performans Status and age, mechanical ventilation was significantly associated with mortality whatever the day of initiation with OR ranging from 1.48 [1.01–2.16] at Day 0 to 16.44 [8.37–32.3] for those ventilated after day 3. As shown, the risk for mortality increased along time. This suggests that delayed intubation could worsen prognosis.

## DISCUSSION

In an era of controversy regarding initial oxygenation and ventilation support strategy to avoid MV in immunocompromised patients with hypoxemic ARF, this large study failed to show any significant association between initial management and need for intubation on the next day. This result is of prime interest as it suggests that restoring oxygenation only represents a single symptomatic treatment.

In the setting of immunocompromised patients with hypoxemic ARF, every study result has an alternate study that reports opposite findings. For instance, although Hilbert *et al.* [18] have found that NIV could reduce intubation rate and mortality, 15 years later this evidence has been challenged by Lemiale *et al*. [13]. Regarding HFNC, a post-hoc analysis from Frat *et al*. [12] suggested that HFNC might be a suitable alternative to standard oxygen or NIV which might even increase MV and mortality rates. On the contrary, the post-hoc analysis from the Lemiale's trial [17] did not find any difference in intubation rate or mortality between HFNC and standard oxygen treatment. Lastly, Mokart *et al*. [14] suggested a significantly improved day-28 survival with the use of combined HFNC–NIV therapy.

Indeed, NIV could be perceived as a harmful noninvasive ventilatory support strategy in hypoxemic ARF. First, as a non-continuous treatment, in-between sessions are exposed to lung derecruitment, oxygenation loss as well as physiological deterioration [19]. Secondly, in patients with high respiratory drive, generous tidal volume exposes patient's lungs to exceeding transpulmonary pressure and the possibility of patient self-inflicted lung injury [20]. This hypothesis is in line with the results from the Frat trial [12], or the LUNGSAFE study [16] that have both reported adverse outcomes from NIV in patients with acute ARF and a Pa02/Fi02 ratio < 150. It is then likely that at least a proportion of patients with hypoxemic ARF cannot be safely managed by NIV. Moreover, any attempt to delay conventional and protective MV in these patients may translate into harm. HFNC has demonstrated its ability to decrease respiratory rate and alleviate respiratory distress [21]. It may then be a protective device in patients with hypoxemic ARF, in line with its reported benefit in the Frat trial [22]. Whether HFNC is as protective as sedation and conventional mechanical ventilation on reducing expiratory tidal volumes and subsequent lung injury is debatable. Trials dedicated to compare HFNC to standard oxygen in immunocompromised patients with ARF, are warranted. Also, studies to compare HFNC or NIV to conventional ventilation in patients with criteria for MV would be timely [23].

The present study has several limitations. First, it was based on three empirical samples and some confounding in the comparison of noninvasive ventilatory support strategies is likely; thus, we used causal inference approaches that are expected to control imbalances in potential confounders between treated groups [24, 25] though we cannot rule out that some residual imbalances have persisted. Second, we retrospectively combined data from two RCTs and one cohort, mostly to increase the external validity of our findings; however, this may have influenced the results by introducing some heterogeneity in patient populations, procedures including applied oxygenation strategies, other patient management and data quality. To check for any trial effect due to case-mix, we compared the cumulative incidences of MV and ICU death between studies, without any evidence of differences. We did not find any significant interactions between included studies and results. Third, the sample sizes of the HFNC groups were somewhat low. However, with 89 patients treated with HNFC alone and 60 with both HFNC and NIV, this study remains the largest in immunocompromised patients. Last, we only describe mortality data according to initial oxygenation/ventilation strategy, without properly assess the effect of oxygenation groups on mortality; such an analysis that would need to handle time-dependent confounders, is certainly of interest for further studies.

Therefore, with the objective to improve outcomes in this high-risk population, trials are needed to better define initial ventilation. A proper evaluation of HFNC versus standard oxygen is required, as noninvasive ventilation has become a potentially harmful strategy in hypoxemic ARF. In addition, patients need to be stratified according to their respiratory drive before choosing the device to keep them under the chopper of noninvasive ventilation strategy.

## CONCLUSIONS

Based on causal inference, this study failed to show any evidence of difference between several noninvasive ventilatory support strategies on mechanical ventilation probability during the first three ICU days in a large cohort of immunocompromised patients with hypoxemic ARF. Randomized trials are warranted in order to clarify initial management of hypoxemic ARF in this population.

## MATERIALS AND METHODS

### Data source

This study is a post hoc joint analysis of three previously published studies conducted in critically ill immunocompromised patients. Details of each study have been described previously [5, 13, 26]. Supplementary Table 1 summarizes their main characteristics. Data reported in Tables 1–3 were prospectively collected.

In all studies, hypoxemic ARF was defined by tachypnea (respiratory frequency > 30/min), respiratory distress, labored breathing, oxygen saturation less than 90% or PaO2 less than 60 mmHg on room air and need for > 6l min of standard oxygen without hypercapnia (paCO2 > 50 mmHg). The decision to perform endotracheal intubation was left to clinicians in charge at each ICU. The appropriate ethics committees approved each individual study. Immunosuppression was defined by a hematological malignancy, a solid tumor requiring treatment since less than 5 years, long term (>3 months) or high dose (>1 mg/kg) steroids, any other immunosuppressive drug, solid organ transplant or primary immune deficiency.

### Selection of the study population

Because NIV is a medically recommended practice in acute pulmonary edema and COPD exacerbation (i.e., acute hypercapnic respiratory failure), we excluded patients with these conditions at ICU admission. Also, only patients with ARF developing within 48 hours of ICU admission were included in this study. All patients were admitted without treatment-limitation decisions at ICU admission, including do-not-intubate order. Last, patients intubated the same day than ICU admission were not included given we focused on predicting the need for intubation on the coming day, and not discriminate among ICU patients of those who will be intubated or not. This last criterion could be seen as hampering external validity to this study given one cannot exclude that those excluded patients may have been administered NIV or HFNC in the same day before intubation. However, NIV and HFNC failure are a source of concern when they are used for more than 3 days [27, 28].

The flow chart of the study is displayed in Supplementary Figure 1.

### Study outcomes and exposures

The main study end-point was the need for MV on the coming day, that is, on day *d*+1, among patients at risk (alive, free of MV and still in the ICU), at day *d*. We restricted ourselves to the first three ICU days (*d* = 0, 1, 2, where *d = 0* defines the day of ICU admission, day 1, 2, etc. were subsequent 24 hours periods) because the vast majority of MV were expected to occur in these first days [13, 17]. Secondary outcome was death within 28 days of ICU admission.

Two daily oxygenation and ventilation strategies were considered successively. First, we only distinguished NIV versus oxygen therapy regardless of the oxygenation device (standard oxygen or HFNC), where NIV defined the exposure of interest, using logistic regression to predict treatment assignment. Secondly, we considered four groups of oxygenation and ventilation strategies, distinguishing first among NIV patients those administered NIV alone and those receiving NIV associated with HFNC, patients receiving HFNC alone and those with standard oxygen therapy alone.

### Statistical analysis

All data are presented as median [interquartile range, IQR] for quantitative variables and frequencies (percentages) for qualitative variables. A nonparametric estimator of the sub distribution function estimated the cumulative incidence of MV requirement in the first 7 days of ICU, taking into account the competing deaths or ICU discharges, all free of MV. The crude conditional probability of MV at day *d*+1 for the first three days of ICU stay, overall and according to the noninvasive oxygenation strategy, was computed.

We first addressed the question of how MV requirement would differ if all the subjects actually treated by NIV were administered the oxygen therapy, when all observed confounders were equally distributed among those two groups. Propensity score (PS) weighted analysis was thus used as detailed in the Supplementary Data. Resulted weighted datasets defined so-called “pseudo-population” at each day, were analyzed using a weighted logistic regression that allowed taking into account the effect of time-varying exposure on the outcome (MV on the coming day or not) based on the weighted dataset. Then, after pooling individual patient data for the three days, marginal model applied with further adjustment on the day of exposure. Effects were summarized as odds ratio (ORs) with respective 95% confidence intervals (95 CIs).

Analyses were rerun to assess the effect of exposure, when considering the 4 strategies separately.

Sensitivity analyses also investigated evidence that treatment effect varies substantially among different subsets of patients, defined by the study and the main diagnosis of ARF, based on the Gail and Simon statistics [29].

To illustrate Day-28 mortality according to the delay since ICU admission and MV initiation, we used logistic regression at each day to provide OR for mortality (adjusted on age, Performans Status and SOFA at admission).

All tests were two-sided at the 0.05 significance level. Data management and statistical analyses were performed using R statistical packages (online at https://www.R-project.org).

## SUPPLEMENTARY MATERIALS FIGURES AND TABLES





## Abbreviations

ARF: Acute respiratory failure
ICU: Intensive care unit
IQR: Interquartile range
IMV: Invasive ventilation
HFNC: High Flow Nasal Cannula therapy alone
NIV: Noninvasive ventilation
OR: Odds ratio
PS: Propensity score
SOFA: Sequential Organ Failure Assessment
95CIs: 95% confidence intervals.
