# Supplementary material for: Oxygenation/non-invasive ventilation strategy and risk for intubation in immunocompromised patients with hypoxemic acute respiratory failure
Source: Oncotarget. 2018 Sep 14;9(72):33682–93. doi: 10.18632/oncotarget.26069 (PMC6154743; doi:10.18632/oncotarget.26069)
Supplement: Supplementary file 1 [file oncotarget-09-33682-s001.pdf]

Oxygenation/non-invasive ventilation strategy and risk for intubation in immunocompromised patients with hypoxemic acute respiratory failure

SUPPLEMENTARY MATERIALS

MATERIALS AND METHODS

Estimation of PS

It consisted at each day (from ICU admission until day +2), to estimate the probability to receive the oxygenation strategy the patient actually received was computed for each patient, as follows. PS models consisted in logistic regression model including potential confounders, namely baseline characteristics (age, gender, underlying disease, respiratory comorbidities, allogenic stem cell transplantation, performance status over 2 in the previous 2 months), covariates known to be linked with mortality or intubation in cancer patients (SOFA score at day, number of quadrants involved on chest X-ray, hemodynamic and neurologic SOFA score over 2), physiologic parameters (neutropenia, pH, partial pressure of arterial oxygen at day (min-max), maximal respiratory rates) and other potential confounders (ARF etiology). In order to incorporate the history of patient status, the PS models at day +1 and +2 also included the physiologic parameters measured on the day before (i.e. day minus 1). Missing values of covariates were handled by multiple imputations with chained equations, based on M = 30 imputed complete datasets [1, 2]. Individual propensity scores were averaged over the M values, as recommended for its potential to produce substantial bias reductions [3]. Different metrics were used to check whether balance has been achieved, based on the comparison of these metrics before and after weighting at each landmark.

Estimation of IPTW

In order to estimate and compare the causal effect of daily respiratory management strategy on the probability of intubation in the coming day, we computed inverse probability of treatment weights (IPTW) using propensity-score (PS) [4]. This approach aims at providing causal estimate of treatment effect, by creating a new sample in which the distribution of measured baseline covariates is independent of treatment assignment [5]. Our quantity of interest was the Average Treatment Effect on the Treated (ATT) that addresses the question of how outcomes would differ if the subjects who were actually treated were given the other choice. We used unstabilized weights given benefits of stabilized weights for dynamic marginal structural model (MSM) has been considered uncertain [6]. Thus, treated subjects receive a weight of 1, while untreated subjects receive a weight of PS/(1-PS).

Two treatment exposures (that is, of non-invasive oxygenation strategies) were considered over time successively. First, each day, we only distinguished NIV versus oxygen therapy regardless the device (standard oxygen or HFNC), where NIV defined the exposure of interest, using logistic regression to predict treatment assignment. Secondly, we considered four groups of non-invasive strategies, distinguishing among NIV patients those administered NIV alone and those receiving NIV associated with continuous administration of oxygen through HFNC, patients receiving HFNC alone and those with standard oxygen therapy alone. As proposed by McCaffrey *et al*. [7], we used Generalized Boosted Model (GBM) for estimation of the IPWT. GBM estimation is a non parametric machine learning technique which used an iterative process with multiple regression trees to take into account nonlinear relationships between pretreatment covariates and treatment assignment [8, 9]. Standard oxygen strategy was defined as the reference group, so that the other three strategies (NIV, HFNC alone or HFNC+NIV) appeared those of potential interest to be compared with the standard. For each comparison, the PS algorithm was run separately with a number of 8,000 iterations in order to optimize the balances.

Assumption checking

We computed for each covariate standardized mean difference, also referred as to the absolute standardized mean difference or the population absolute standardized bias (PSB) [10], considering that a standardized difference below 10% or 20% [7] was an acceptable threshold indicative of negligible imbalance. As an overall balance

measure, we then computed the C-statistic derived from the ROC curve from the PS model on the weighted sample for the two-treatment exposure model [11], and the Kolmogorov-Smirnov (KS) statistic for the four-treatment exposure model [10]. We also assessed the positivity assumption by an examination of the weights values and distribution. Otherwise, to avoid extreme weights due to near violations of the positivity assumption, we truncated weights at the 90th percentile (i.e., any weight larger than the 90th percentile was assigned to the 90th percentile) [12].

Analysis of the treatment effect

Model 1: Oxygen alone vs. Non invasive ventilation

Propensity score and inverse probability weighted based analysis

Covariates balance before and after weighting is reported in Supplementary Figure 5A. As expected, the imbalances before weighting as measured by the standardized mean differences were decreased after weighting, all below the threshold of 10%. The reduced balance in covariates was also illustrated by the values of the C-statistic for the three final models that were 0.566, 0.558 and 0.526, respectively. The mean of the weights in the final MSM was 0.78 with a maximum value of 5.8 and a minimum value of 0.06 (Supplementary Figures 6 and 7).

Model 2: Standard oxygen therapy alone vs. Non invasive ventilation and high flow nasal cannula

Propensity score analysis and Inverse Probability Weighted based analysis

Supplementary Figure 5B displays the standardized mean differences on the original sample and after weighting. Graphical examination suggests that the IPTW has created a similar distribution of measured covariates between treated and control subjects, though some imbalances were still above 0.2. Supplementary Figure 8 shows the different weights according to the oxygenation strategy in the MSM. The mean value of weights was 1.14 with extreme values ranging from 0.02 to 15.7. Although the mean value was close to one, extreme values likely reflect the limited sample size of the NIV with HFNC group.

REFERENCES

1. White IR, Royston P, Wood AM. Multiple imputation using chained equations: issues and guidance for practice. Stat Med. 2011; 30:377–99.
2. Vesin A, Azoulay E, Ruckly S, Vignoud L, Rusinovà K, Benoit D, Soares M, Azeivedo-Maia P, Abroug F, Benbenishty J, Timsit JF. Reporting and handling missing values in clinical studies in intensive care units. Intensive Care Med. 2013; 39:1396–404.
3. Mitra R, Reiter JP. A comparison of two methods of estimating propensity scores after multiple imputation. Stat Methods Med Res. 2016; 25:188–204.
4. Rosenbaum PR, Rubin DB. The central role of the propensity score in observational studies for causal effects. Biometrika. 1983; 70:41–55.
5. Lunceford JK, Davidian M. Stratification and weighting via the propensity score in estimation of causal treatment effects: a comparative study. Stat Med. 2004; 23:2937–60.
6. Cain LE, Robins JM, Lanoy E, Logan R, Costagliola D, Hernán MA. When to start treatment? A systematic approach to the comparison of dynamic regimes using observational data. Int J Biostat. 2010; 6:18.
7. McCaffrey DF, Griffin BA, Almirall D, Slaughter ME, Ramchand R, Burgette LF. A tutorial on propensity score estimation for multiple treatments using generalized boosted models. Stat Med. 2013; 32:3388–414.
8. McCaffrey DF, Ridgeway G, Morral AR. Propensity score estimation with boosted regression for evaluating causal effects in observational studies. Psychol Methods. 2004; 9:403–25.
9. Griffin BA, Ramchand R, Almirall D, Slaughter ME, Burgette LF, McCaffery DF. Estimating the causal effects of cumulative treatment episodes for adolescents using marginal structural models and inverse probability of treatment weighting. Drug Alcohol Depend. 2014; 136:69–78.
10. Ridgeway G, McCaffrey D, Morral A, et al. Toolkit for Weighting and Analysis of Nonequivalent Groups: A tutorial for the twang package. R Vignette RAND. 2015.
11. Franklin JM, Rassen JA, Ackermann D, Bartels DB, Schneeweiss S. Metrics for covariate balance in cohort studies of causal effects. Stat Med. 2014; 33:1685–99.
12. Shepherd BE, Liu Q, Mercaldo N, Jenkins CA, Lau B, Cole SR, Saag MS, Sterling TR. Comparing results from multiple imputation and dynamic marginal structural models for estimating when to start antiretroviral therapy. Stat Med. 2016; 35:4335–51.

Supplementary Table 1: Characteristics of included studies. See Supplementary_Table_1

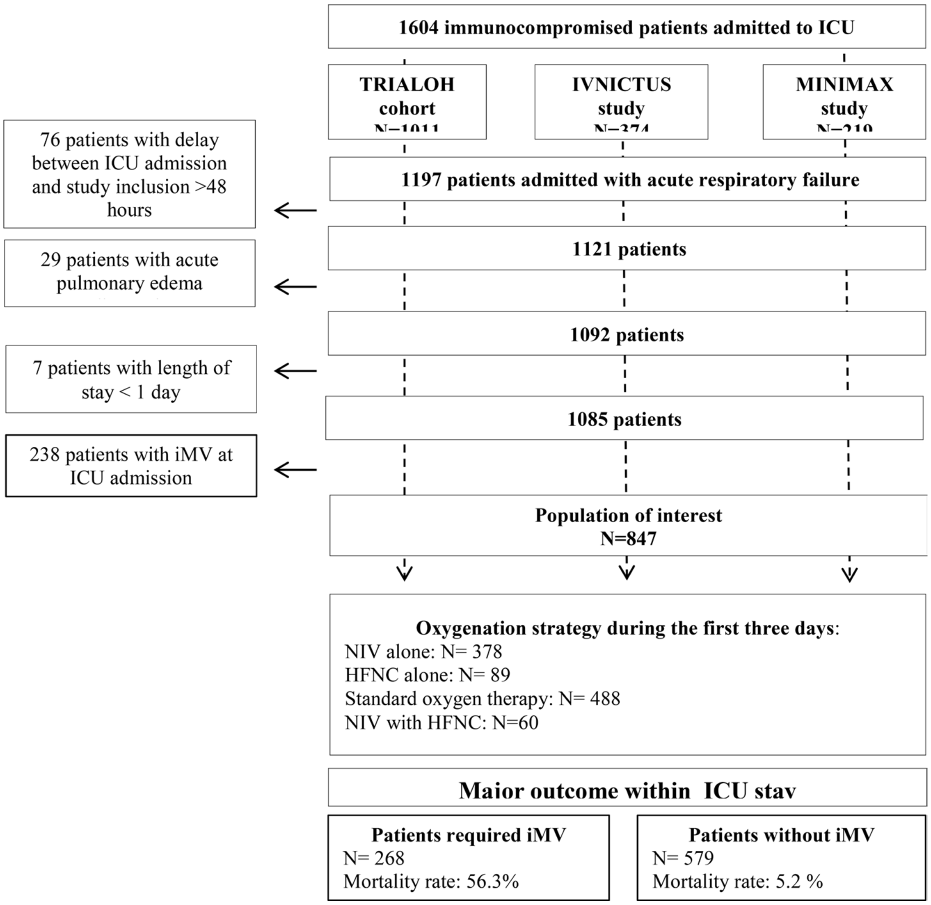

Supplementary Figure 1: Flow chart of the study. ARF: acute respiratory failure, ICU: intensive care unit, NIV: Noninvasive ventilation, HFNC: high flow nasal cannula, iMV: invasive mechanical ventilation.

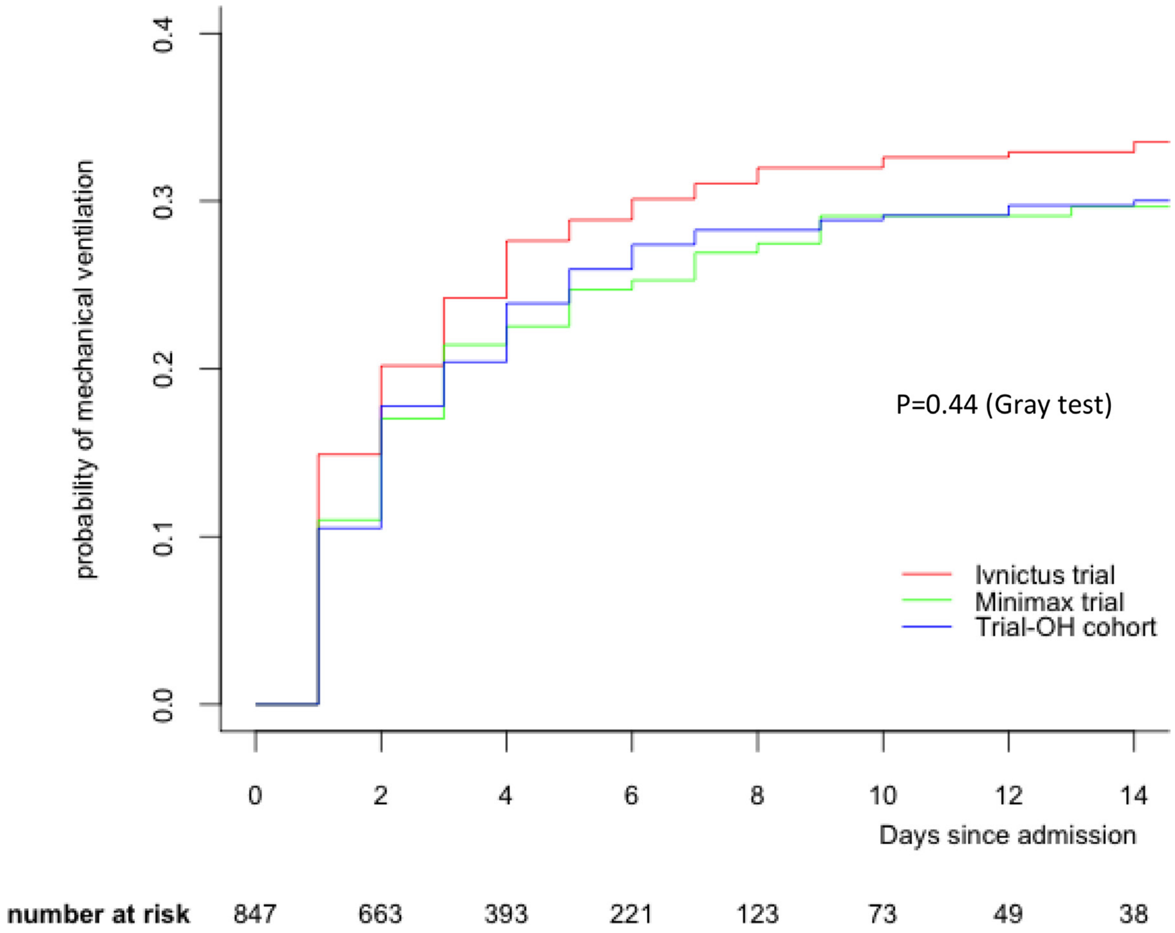

Supplementary Figure 2: Cumulative incidence of intubation over 14 days.

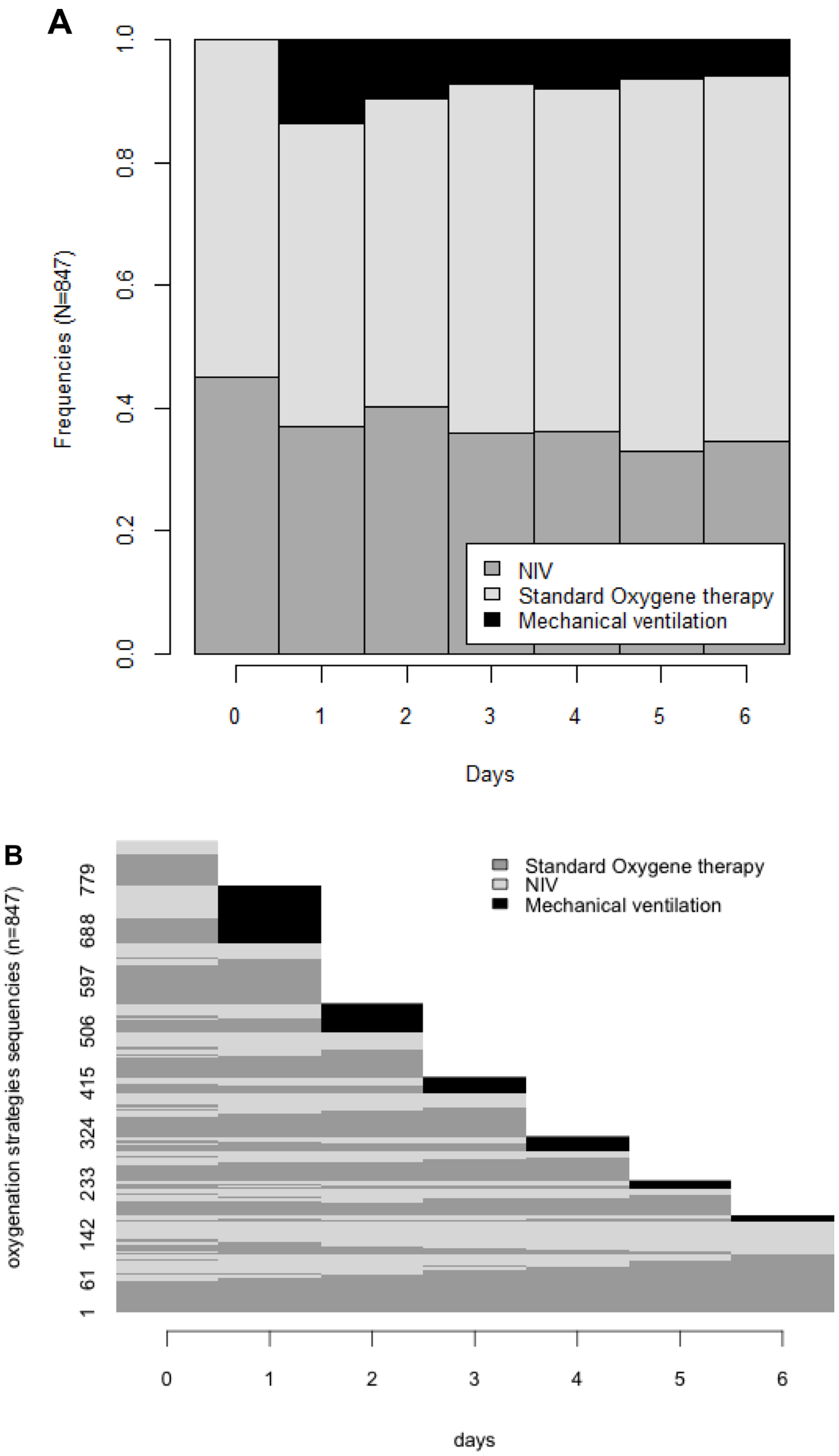

Supplementary Figure 3: Oxygenation strategies within the first 7 days of the ICU course distinguishing NIV and oxygen therapy - Model 1- State distribution (**A**) and Oxygenation strategies sequences (**B**). NIV: Noninvasive ventilation.

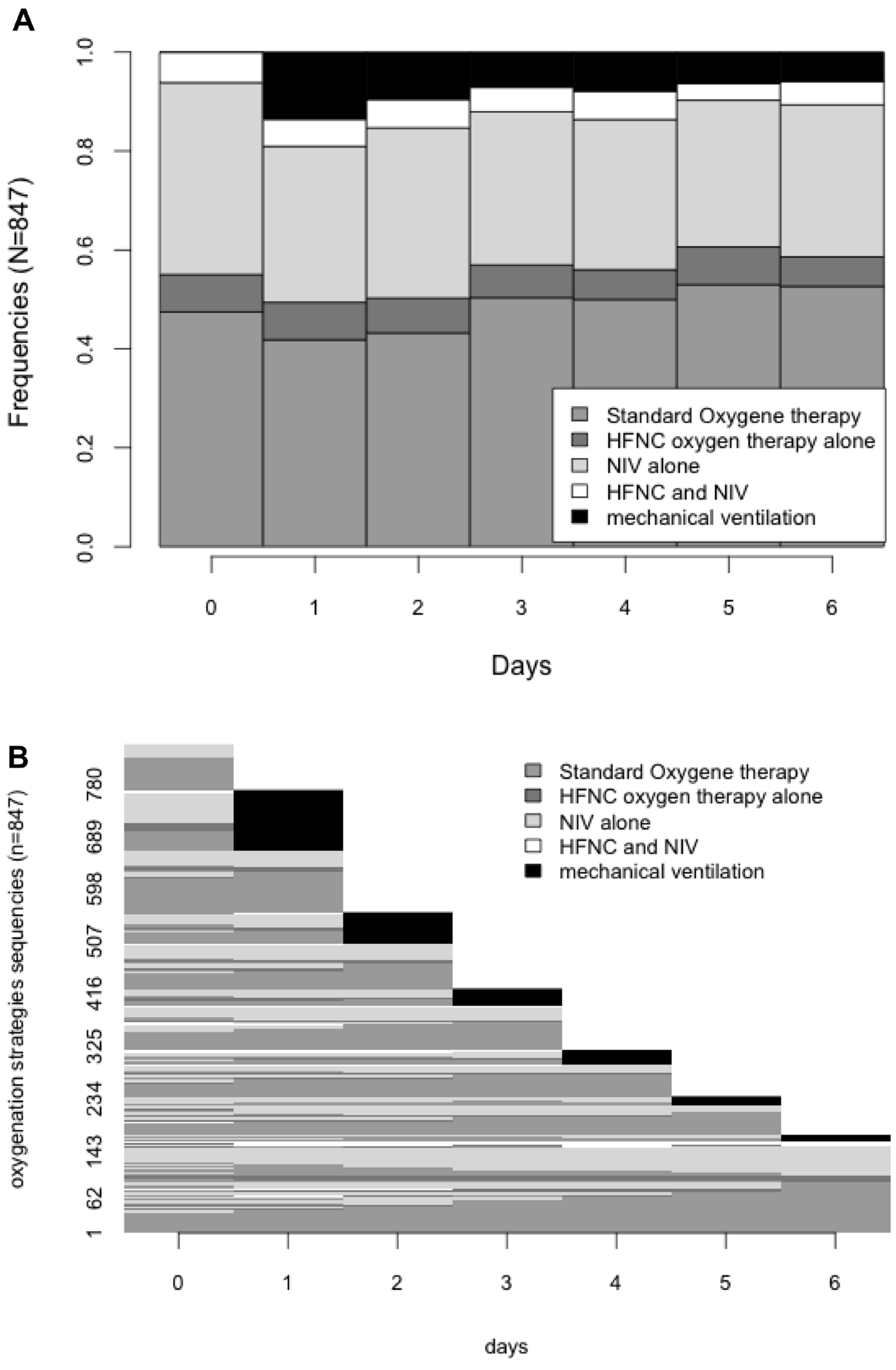

Supplementary Figure 4: Oxygenation strategies within the first 7 days of the ICU course distinguishing NIV, continuous administration of oxygen through HFNC, standard oxygen therapy and NIV associated with HFNC - Model 2- State distribution (**A**) and Oxygenation strategies sequences (**B**). NIV: Noninvasive ventilation, HFNC: high flow nasal cannula.

A

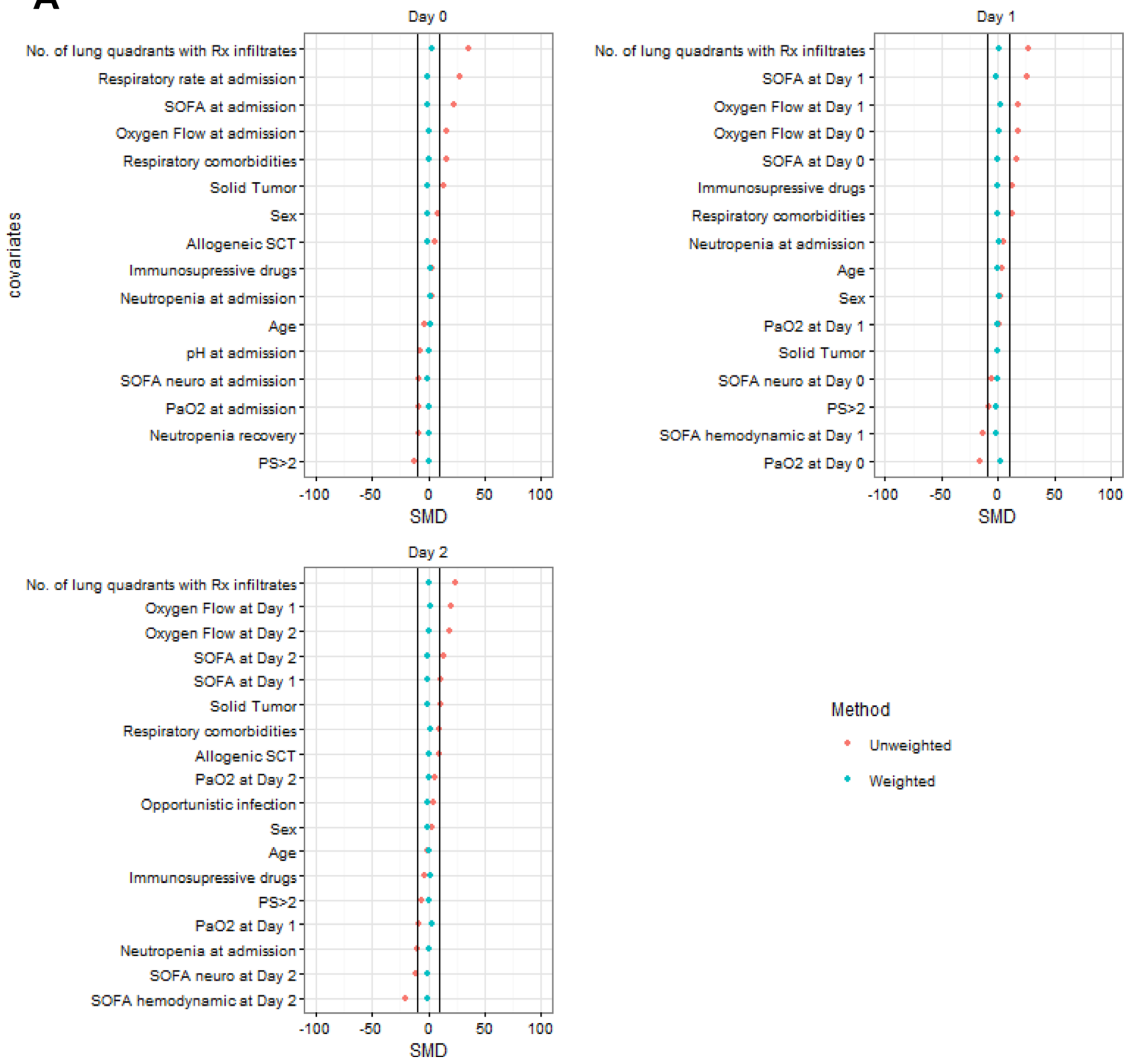
Day 0
Day 1
Day 2
covariates
No. of lung quadrants with Rx infiltrates
Respiratory rate at admission
SOFA at admission
Oxygen Flow at admission
Respiratory comorbidities
Solid Tumor
Sex
Allogeneic SCT
Immunosupressive drugs
Neutropenia at admission
Age
pH at admission
SOFA neuro at admission
PaO2 at admission
Neutropenia recovery
PS>2
SOFA at Day 1
Oxygen Flow at Day 1
Oxygen Flow at Day 0
SOFA at Day 0
PaO2 at Day 1
SOFA neuro at Day 0
SOFA hemodynamic at Day 1
PaO2 at Day 0
Oxygen Flow at Day 2
SOFA at Day 2
Allogenic SCT
PaO2 at Day 2
Opportunistic infection
SOFA neuro at Day 2
SOFA hemodynamic at Day 2
-100 -50 0 50 100
SMD
Method
Unweighted
Weighted

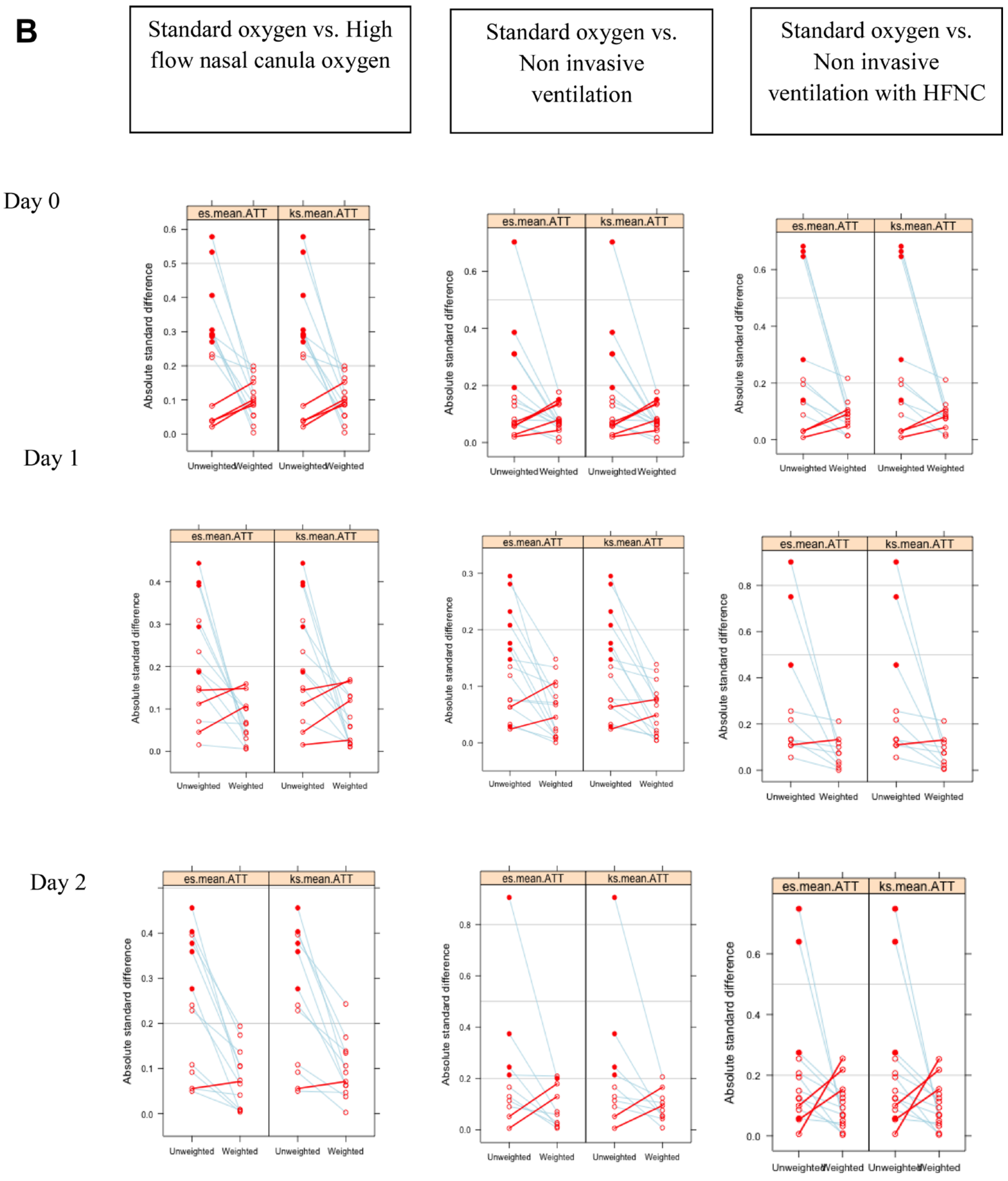

Supplementary Figure 5: Standardized mean difference in main covariates between treatment groups in unweighted and weighted sample on the first three days of ICU stay- in the first model (**A**) and the model 2 (**B**).

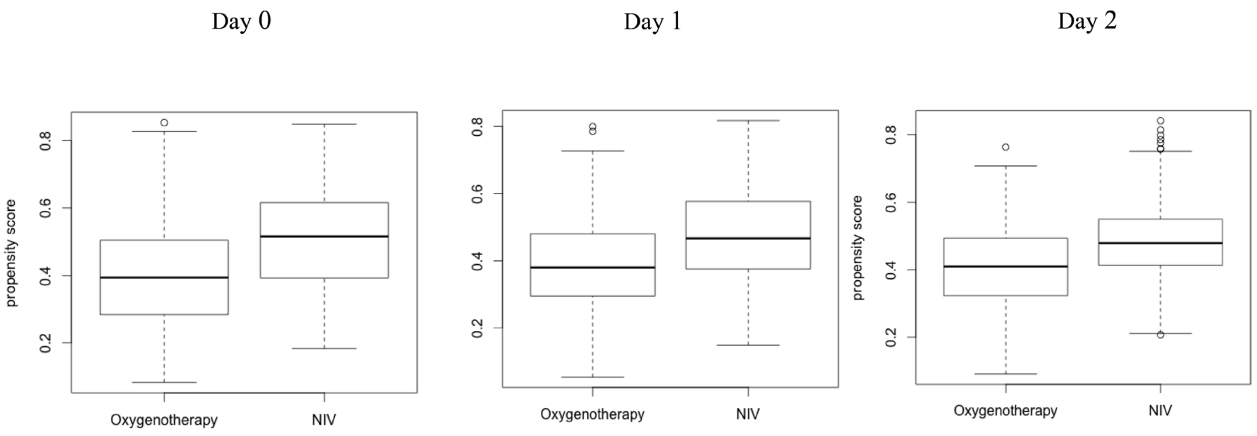

Supplementary Figure 6: Distribution of treatment probability according to baseline oxygenation strategy (day 0 to day 2).
NIV: Noninvasive ventilation.

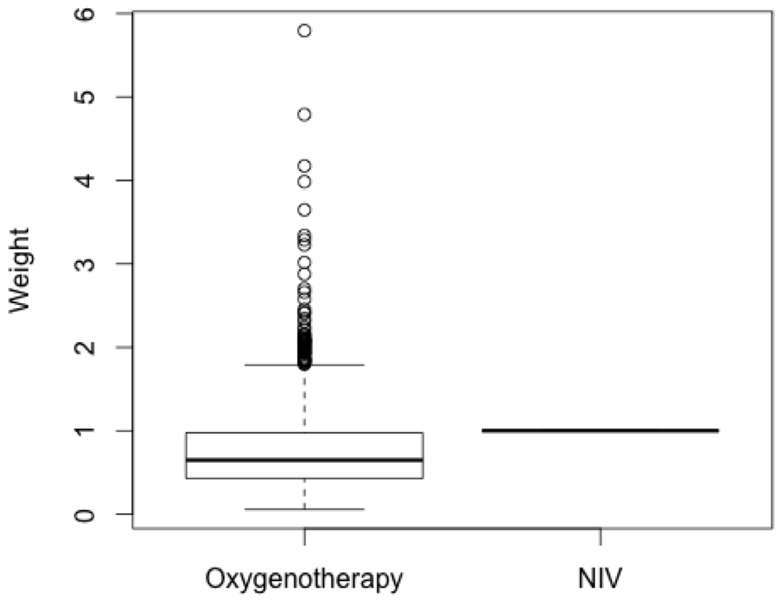

Supplementary Figure 7: Distribution of weighs in the final MSM (model 1). NIV: Noninvasive ventilation.

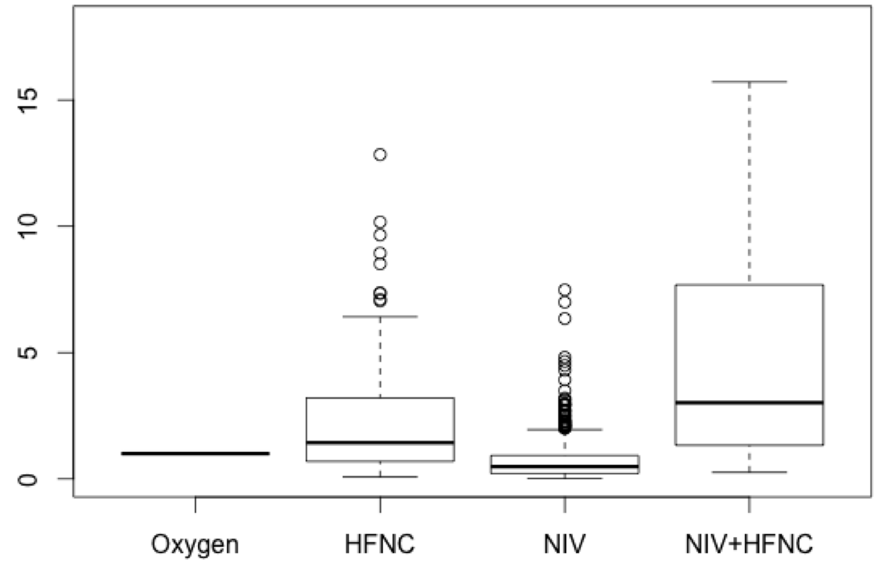

Supplementary Figure 8: Distribution of weights according to the oxygenation strategy (Model 2). Oxygen: standard oxygen group, HFNC: high flow nasal cannula, NIV: non invasive ventilation, NIV+HFNC: non invasive ventilation with high flow nasal cannula.
