# Supplementary material for: Oxygenation/non-invasive ventilation strategy and risk for intubation in immunocompromised patients with hypoxemic acute respiratory failure
Source: Oncotarget. 2018 Sep 14;9(72):33682–93. doi: 10.18632/oncotarget.26069 (PMC6154743; doi:10.18632/oncotarget.26069)
Supplement: Supplementary file 2 [file oncotarget-09-33682-s002.docx]

Supplementary Table 1: Characteristics of included studies

|  | **TRIAL-OH study** | **MINIMAX trial** | **IVNICTUS trial** |
| --- | --- | --- | --- |
| **Source** | Azoulay E. et al. | Azoulay E. et al. | Lemiale V. et al |
| **Year of publication** | 2013 | 2010 | 2015 |
| **Number of patients** | 1011 | 219 | 374 |
| **Period of recruitment** | Jan. 2010- May 2011 | Sept. 2005-Nov.2007 | Aug. 2013-Jan. 2015 |
| **Study centers^a^** | 17 centers in France and Belgium | 16 centers in France | 28 centers in France and Belgium |
| **Study design** | Prospective observational cohort | Randomized Controlled trial  (open label) | Randomized Controlled trial  (open label) |
| **Population** | Patients with hematologic malignancies admitted in ICU  (acute respiratory failure: n=632) | Critically ill cancer patients with acute respiratory failure | Immunocompromised patient admitted in ICU with acute respiratory failure |
| **Study intervention** | None | Intervention arm: FO-BAL on day 1 for ARF diagnosis (n=113)  Control arm: non invasive testing for ARF diagnosis (n=106) | Intervention arm: oxygenation strategy by NIV (n=191)  Control arm: standard oxygen or HFNC (n=183) |
| **Type of randomization** | Not applicable | 1: 1 ratio  Randomization stratification: center | 1: 1 ratio  Randomization stratification:  Center, oxygen flow rate at randomization (> or ≤9 L/min), cause of immunosuppression |
| **Inclusion criteria** | - Hematologic malignancies - Admission required in ICU for any reason - 18 years or older | - Solid cancer or hematologic malignancies - ARF^b^ - 18 years or older | - Immune deficiency ^c^ - ARF^b^ - 18 years or older |
| **Exclusion criteria** | - Complete cure of the malignancy for more than 5 years - ICU admission only to maximize safety of a procedure - Age younger than 18 years | - Contraindications to FO-BAL (coma, shock, or SaO2 < 90% while breathing oxygen through a Ventury mask) - Cardiogenic pulmonary edema - ARF due to known causes - Endotracheal mechanical ventilation - Treatment-limitation decisions | - Contraindications to NIV - Hypercapnia - Need for immediate invasive mechanical ventilation - Cardiogenic pulmonary edema - Epinephrine or norepinephrine > 0.3 μg/kg/min - Ongoing myocardial infarction or acute coronary syndrome - Glasgow Coma Scale score <13 - Do-not-intubate decision - Long-term oxygen therapy - Postoperative ARF |
| **Primary outcome** | Vital status at hospital discharge | Intubation rate and mortality | All-cause mortality within  28 days after randomization. |
| **Other outcome** | Evaluate benefits of starting life-supporting interventions late during the ICU stay or using life-supporting interventions for prolonged periods | Evaluate non invasive strategy on:  diagnostic, 28-day mortality, ICU acquired infections, number of antibiotic-free days, and infection by multiresistant bacteria | - Endotracheal intubation - SOFA score on day 3 - ICU-acquired infections - Mechanical ventilation duration - ICU lengths of stay |
| **Baseline definition** | Time of inclusion | ICU admission (day 1) | Time of randomization |
| **Data collection** | Prospectively by investigators:  Days 1-2 -3-5-7-14-21-28  Month 6 | Prospectively by investigators:  Days 1-2 -3-5-7-14-21-28 | Prospectively by investigators:  Days 1 to 7, Days-14-28  Month 6 |
| **Intubation rate**  **N (%)** | 484 (47.9) | 82 (37.4) | 155 (41.4) |
| **Mortality rate, %** | 39.3 (in-hospital mortality) | 31 (Day-28 mortality) | 35.7 (Day-28 mortality) |

ICU: intensive care unit, ARF: acute respiratory failure, NIV: non-invasive ventilation, HFNC: high flow nasal cannula, FO-BAL: Fiber optic bronchoscopy with bronchoalveolar lavage, SOFA: Sequential Organ Failure Assessment score.

^a^ Common centers: 13 IVNICTUS trial /MINIMAX trial, 12 MINIMAX trial / TRIAL-OH study, 16 TRIAL-OH study/IVNICTUS trial

^b^ Defined as: oxygen saturation less than 90% or PaO2 less than 60 mm Hg on room air combined with severe dyspnea at rest with inability to speak in sentences or respiratory rate greater than 30 breaths per minute or clinical signs of respiratory distress

^c^Defined as: hematologic malignancy or solid tumor (active or in remission for less than 5 years), solid organ transplant, long-term (>30 days) or high-dose (>1 mg/kg/d) steroids, or any immunosuppressive drug taken in a high dosage or for more than 30 days
